# Unsupervised inference of implicit biomedical events using context triggers

**DOI:** 10.1186/s12859-020-3341-0

**Published:** 2020-01-28

**Authors:** Jin-Woo Chung, Wonsuk Yang, Jong C. Park

**Affiliations:** School of Computing, KAIST, 291 Daehak-ro, Yuseong-gu, Daejeon, Republic of Korea

**Keywords:** Biomedical event extraction, Unsupervised inference, Cross-sentence relations, Bacteria, Biotope, Natural language processing, Text mining

## Abstract

**Background:**

Event extraction from the biomedical literature is one of the most actively researched areas in biomedical text mining and natural language processing. However, most approaches have focused on events within single sentence boundaries, and have thus paid much less attention to events spanning multiple sentences. The Bacteria-Biotope event (BB-event) subtask presented in BioNLP Shared Task 2016 is one such example; a significant amount of relations between bacteria and biotope span more than one sentence, but existing systems have treated them as false negatives because labeled data is not sufficiently large enough to model a complex reasoning process using supervised learning frameworks.

**Results:**

We present an unsupervised method for inferring cross-sentence events by propagating intra-sentence information to adjacent sentences using context trigger expressions that strongly signal the implicit presence of entities of interest. Such expressions can be collected from a large amount of unlabeled plain text based on simple syntactic constraints, helping to overcome the limitation of relying only on a small number of training examples available. The experimental results demonstrate that our unsupervised system extracts cross-sentence events quite well and outperforms all the state-of-the-art supervised systems when combined with existing methods for intra-sentence event extraction. Moreover, our system is also found effective at detecting long-distance intra-sentence events, compared favorably with existing high-dimensional models such as deep neural networks, without any supervised learning techniques.

**Conclusions:**

Our linguistically motivated inference model is shown to be effective at detecting implicit events that have not been covered by previous work, without relying on training data or curated knowledge bases. Moreover, it also helps to boost the performance of existing systems by allowing them to detect additional cross-sentence events. We believe that the proposed model offers an effective way to infer implicit information beyond sentence boundaries, especially when human-annotated data is not sufficient enough to train a robust supervised system.

## Background

The rapidly growing volume of biomedical literature published every year has called for efficient tools to collect important information of interest from unstructured text. Biomedical natural language processing has played an important role in addressing this need, where researchers have addressed various tasks and applications, such as named entity recognition, sentence classification, uncertainty detection, event or relation extraction, and question answering [1, 2]. In particular, extraction of events and relations about biomedical concepts has recently received much attention through community challenges and shared tasks on collecting various types of biomedical information. For example, researchers have been interested in protein-protein interaction [3], disease-related biological processes [4], gene-related processes [5, 6], drug-drug interaction [7], and chemical-induced disease relations [8], and chemical-protein relations [9]. Event/relation information of interest in such tasks is usually expressed and detected on a sentence level, where a single sentence is assumed to cover complete pieces of information to be extracted. Most benchmark datasets released by organizers of these challenges contain gold-standard annotations of sentence-level information. This has promoted subsequent studies on using syntactic dependencies among mentions about biomedical entities, which has been considered as an important aspect of sentence-level event extraction [10–14].

More recently, researchers have begun to pay attention to events that are not clearly signaled by syntactic dependencies. In particular, extracting cross-sentence events, where relevant entities are found across multiple sentences, is considered challenging because of the lack of clear linguistic evidence for how entities mentioned in different sentences are associated to form a single event. For this reason, few studies have looked into this issue [15–20].

The Bacteria-Biotope event (BB-event) subtask of the BioNLP Shared Task 2016 [21] presents an example of such tasks. The goal is to detect binary relations, called the Live_In events, between bacteria and their physical locations, whose mentions are pre-annotated in a given document by humans. The goal is similar to that of other event and relation extraction tasks in many ways, but the challenge is in cross-sentence events, which amount to 27% (237/890) of the entire event instances. Participating teams employed methods of traditional event extraction, whose systems are trained to distinguish between positive and negative events using various machine learning algorithms such as support vector machine (SVM), neural networks, or their combination [21]. However, most of them did not detect cross-sentence events at all, and just treated them as false negatives, mainly because attempts to detect them led to worse performance due to loss of precision without improvement in recall. Team VERSE, which ranked first in the shared task using support vector machine and linguistic features, reported that they conducted experiments with various settings but achieved the best result only when all the cross-sentence events are ignored (i.e., treated as false negatives) [22]. Task organizers also considered cross-sentence event extraction as a major challenge to the overall BB-event task [21]. Some follow-up studies have been conducted since then, and updated the state-of-the-art performance with diverse types of neural networks built over syntactic dependency paths between entities. Examples include bidirectional long-short term memory networks [23], gated recurrent unit networks [24], and recursive and recurrent neural networks [20]. However, they either did not extract cross-sentence events at all, or achieved the best results only when cross-sentence events are ignored. This is probably because labeled examples of cross-sentence events are much fewer than those of intra-sentence ones, making it difficult to train a supervised system with high-dimensional representations. These research results highlight important challenges to cross-sentence event extraction in this shared task.

Although recent studies addressed the extraction of cross-sentence events in other tasks, they are markedly different from the BB-event task for the following reasons: They either (1) targeted relatively coarse-grained information such as concept-level (e.g., MeSH ID) or document-level annotations, as found in Biocreative V Chemical-Disease Relation (CDR) datasets [8], (2) used curated knowledge or combinations of different knowledge bases, such as Comparative Toxicogenomics Database [15, 16, 25], or (3) relied on relatively large training data (e.g., 500 documents in the CDR datasets vs. 61 in the BB-event datasets), further expanded with curated databases and distant supervision, on which high-dimensional network or graph-based systems can be trained [15, 17, 18]. By contrast, the BB-event task targets fine-grained mention-level relations with minimal training data, and no task-specific curated knowledge bases are available. Recent research results suggest that supervised systems based on high-dimensional representation learning would not be very effective at addressing the data sparsity issue in cross-sentence events in the BB-event datasets [20, 21, 23, 24].

In this paper, we propose an inference system to detect cross-sentence events, without relying on supervised learning on annotated data or any curated knowledge base. The main idea is to propagate entity information found in a sentence over its adjacent sentences through contextual information. More specifically, if an entity is mentioned in a sentence, we note that it is possible to find expressions in its adjacent sentences that imply the *presence* of that entity. Then, by using these expressions, we convert cross-sentence relations into intra-sentence relations. We call these expressions *context triggers* because they trigger a contextual window where the presence of an entity is naturally acknowledged by context, even when it is not mentioned.

The two consecutive sentences in Example 1 taken from the BB-event training data demonstrate how context triggers signal the presence of an entity mentioned in an adjacent sentence, and how they are used to extract the cross-sentence event Lives_In<*K. kingae*, *pharyngeal*>. In the example, BACTERIA and LOCATION refer to bacteria and location mentions, respectively, pre-annotated by humans and given in the training data. Example 1.

- *None of the colonized children experienced an invasive* [*K. kingae*] **BACTERIA***infection*.
- *The prevalence of* [*pharyngeal*] **LOCATION***carriage among surgical patients was 8.0%, and...*

Here, considering the meaning of the second sentence, *carriage* involves some bacteria entity mentioned around the sentence, which, in this case, would be the closest one, *K. kingae*, in the preceding (first) sentence. Once we speculate that *carriage* is associated with *K. kingae*, then the next step is to check whether the intra-sentence relation between *carriage* and *pharyngeal* holds or not. This would be much easier than to directly determine whether the relationship between *K. kingae* and *pharyngeal* holds or not. Since *carriage* and *pharyngeal* have a syntactically close relationship (i.e., *pharyngeal* is a direct modifier of *carriage*), we infer the cross-sentence relation between *K. kingae* and *pharyngeal*, using the transitivity *K. kingae* →*carriage* →*pharyngeal*.

There are a number of expressions of this kind in the literature which indicate bacteria-related biomedical actions, responses, or functions that involve physical locations, such as *carriage*, *transmission*, *resistance*, and *infection*. We note that they can be considered as a type of indirect anaphor for bacteria entities mentioned in a given document. This means that they can also be used as contextual clues to cross-sentence inference, as shown in the example above. We also note that this is similar to how humans recognize implicit information across sentences. In order to perform inference of this kind, it is important to see the kind of expressions that can be used as context triggers and the way to collect them. Our intuition is that they are likely to be found in sentences with certain types of syntactic construction and bacteria mentions. For example, the syntactic construction “{noun} of BACTERIA in LOCATION” restricts the semantics of “{noun}” to a bacteria-related process with respect to a location, such as “*antimicrobial ****resistance**** of*[*Campylobacter*] **BACTERIA***in* [*UK retail poultry*] **LOCATION**”. Most important, these expressions can be collected on a larger scale from unlabeled text than from the given training data if we have a list of bacteria names and an access to syntactic dependencies of a given sentence. Note that in this syntactic construction it is not necessary to recognize specific location names such as *UK retail poultry* because the locational preposition *in* already implies the existence of a location (though this preposition can also be used with non-location entities, such as *in 2015*). Once context trigger expressions are identified from a given document, we can use them for cross-sentence inference in the way described above. We explain the overall process in detail with insightful examples throughout the paper.

The experimental results from the official BB-event evaluation benchmark demonstrate that our unsupervised inference system outperforms all the shared task participants including high-dimensional models such as neural networks. It also outperforms state-of-the-art systems when combined with one of the existing supervised systems for intra-sentence event extraction. Our approach can be used not only in an unsupervised manner, but also in a supervised setting. More specifically, context triggers collected from a large amount of unlabeled data can be used as additional features for high-dimensional models to overcome the lack of training signals.

The main idea of this work is inspired by Chung et al. [19] that proposed propagation-based inference of event locations in news articles. They search adjacent sentences for expressions referring to the location of a given event (verb), and use distributional similarities between words to select spatially related events. In contrast to their approach that relies on pre-trained general-purpose distributional similarities for information propagation, we use context trigger expressions that signal the presence of biomedical entities, where they can be collected from a large amount of unlabeled text. We also show the impact of our approach by testing it on standard benchmark datasets in which only a small number of cross-sentence labeled examples are available, and by comparing it with existing approaches.

## Results

In this section, we present experimental data, settings, and results. The overall architecture of the system and specific methods including context triggers are detailed in the “Methods” section.

### Data and settings

We evaluated our models on the official BB-event test data through the online evaluation service provided by the BioNLP-ST 2016 task organizers, where gold-standard reference event annotations in the test data are not accessible to users. The test data consists of 336 bacteria mentions, 757 location mentions, and 340 Lives_In events, where 92 of the events (27.1%) are cross-sentence ones [21]. The performance of the proposed models is assessed by standard evaluation metrics: precision, recall, and F1. They are computed based on the matching similarities between detected events and reference events. The matching similarity of a detected (or reference) event is 1 if the corresponding bacteria-location pair is the same (or equivalent) as any pair in reference (or detected) events. Otherwise, the matching similarity is 0.

We experimented with two different lists of context triggers, created from different data: the training data and the large-scale unlabeled data. We applied the frequency threshold to the triggers collected from the unlabeled data to select only salient triggers. The threshold was empirically set as 300, based on our pilot experiments on the development data. However, for the triggers collected from the training data, we did not apply any frequency threshold, considering their very small number of occurrences. The size of context window for extracting cross-sentence events was also empirically set as [ −3, +3] based on the pilot experiments. This means that only the preceding and following three sentences are considered as within a context window to contain cross-sentence events for a given bacteria mention, provided that the window does not contain any other bacteria mention in it. We thus treated as false negatives all the cross-sentence events that are positioned outside the context window.

We used trigger-based inference as a single method for extracting cross-sentence events, and compared two different methods for extracting intra-sentence events: (1) using the output produced by the VERSE system, and (2) using our syntactic rules and trigger-based inference. As discussed in the “Methods” section, extraction of cross-sentence events was performed on top of intra-sentence extraction. For the VERSE system, we used its updated interface [26] that contains the original VERSE project [22]. Since it does not provide a pre-trained model, we trained a system using its training interface and the same SVM hyper-parameters proposed in their original paper [22]. The trained system achieves the same performance of F1=55.8% as reported in their paper.

### Experimental results

**Context triggers** In the process of collecting context triggers from the PubMed and PMC data, we retrieved a total of 1,450,843 sentences that contain at least one bacteria name, from 106,116 abstracts and 94,682 full-text articles, with 1,660,875 occurrences of bacteria names. We identified 418,889 occurrences of candidate trigger expressions matched by our trigger patterns, and obtained 1250 distinct expressions after lemmatization. By contrast, we collected only 23 distinct trigger expressions from the training data (consisting of 61 abstracts), which are far fewer than those collected from the unlabeled data. We filtered out non-trigger expressions from both of the two collections as explained in the “Methods” section, and applied the frequency threshold to the triggers collected from the unlabeled data. This compile process resulted in 15 triggers for the training data and 47 triggers for the unlabeled data, which we used for the experiments on the test data.

Table 1 shows examples of context triggers compiled from each data, with up to 20 most frequent ones sorted by their frequencies. Not surprisingly, the overall frequency of triggers is much higher in the unlabeled data than in the training data. We found that the collected triggers represent various types of biomedical concepts: event-level concepts such as *infection*, *growth*, *response*, and *survival*; entity-level concepts such as *strain*, *bacteria*, *bacterium*, *organism*, and *microorganism*; disease terms such as *disease*, *bacteremia*, *pathogen*, and *enteritis* (some of them are not shown in the table). All of these triggers imply the presence of bacteria with different degrees in biomedical articles. We also found that all the triggers compiled from the training data are included in the triggers compiled from thex unlabeled data.

**Table 1:** Comparison of context triggers compiled from the BB-event training data and the large-scale unlabeled data

No	Compiled from the training data		Compiled from the unlabeled data	
	Context trigger	Frequency	Context trigger	Frequency
1	isolate	8	strain	21256
2	infection	5	infection	12856
3	strain	5	isolate	9555
4	attachment	3	prevalence	3300
5	adhesion	3	growth	2868
6	bacteremia	2	detection	2621
7	enrichment	2	resistance	2479
8	growth	2	bacteria	1812
9	carriage	2	pathogen	1711
10	resistance	2	culture	1412
11	detection	1	response	1358
12	bacteriophage	1	survival	1275
13	surveillance	1	abundance	1196
14	isolation	1	susceptibility	1161
15	elimination	1	colonization	1015
16			isolation	935
17			transmission	853
18			exposure	846
19			disease	818
20			adhesion	791

They are sorted by their occurrence frequencies in the data

**Performance of intra-sentence and cross-sentence extraction** In order to analyze how well each of the intra-sentence and cross-sentence parts works and how much the latter contributes to the overall performance, we evaluate the two parts separately. We also evaluate the cross-sentence part with complete (i.e., gold-standard) intra-sentence events to assess its pure quality. Note that since the test data do not contain gold-standard reference labels and the evaluation benchmark does not also provide detailed information about how many reference events are correctly extracted, it is not possible to conduct this experiment on the test data. Therefore, we instead use the development data with gold-standard intra-sentence events for this experiment.

Table 2 presents the evaluation results on the development data for three types of events: intra-sentence events, cross-sentence events, and all the events. We achieve F1-scores of 34.7 and 38.1 as the pure cross-sentence extraction performance when using the gold-standard and the extracted intra-sentence events, respectively. The scores themselves seem quite low, but the cross-sentence extraction still maintains or improves the overall performance (i.e., 85.1 →85.1 using the gold-standard events, and 55.8 →58.2 using the extracted events). We consider this as a promising result given the difficulties of extracting cross-sentence events. Note that existing studies also propose methods for cross-sentence extraction for this task, but do not adopt them for the final evaluation because they decrease the overall performance on the test data, according to their papers [20, 22]. In contrast, our cross-sentence extraction improves the overall performance on the test data, to be discussed in the next subsection.

**Table 2:** Comparison of intra-sentence and cross-sentence extraction on the development data

Gold intra-sentence events only						
	Gold	Predicted	Correct	Precision	Recall	F1
Intra	165	165	165	100.0	100.0	100.0
Cross	58	0	0	0.0	0.0	0.0
All	223	165	165	100.0	74.0	85.1
Gold intra-sentence events + our cross-sentence extraction						
	Gold	Predicted	Correct	Precision	Recall	F1
Intra	165	165	165	100.0	100.0	100.0
Cross	58	40	17	42.5	29.3	34.7
All	223	205	182	88.8	81.6	85.0
Our intra-sentence extraction only						
	Gold	Predicted	Correct	Precision	Recall	F1
Intra	165	325	153	47.1	92.7	62.4
Cross	58	0	0	0.0	0.0	0.0
All	223	325	153	47.1	68.6	55.8
Our intra-sentence & cross-sentence extraction						
	Gold	Predicted	Correct	Precision	Recall	F1
Intra	165	325	153	47.1	92.7	62.4
Cross	58	47	20	42.6	34.5	38.1
All	223	372	173	46.5	77.6	58.2

**Overall performance comparison** Table 3 shows the performance of event extraction with the number of intra-sentence and cross-sentence predictions across the proposed and existing models. We compare our models with three top-ranked models of the BB-event shared task participants, and with three recent state-of-the-art models. LIMSI [27] uses SVM with simple surface features such as a bag of tokens in entity mentions. TurkuNLP [28] employs long short-term memory (LSTM) networks with word embeddings and linguistic features taken from shortest dependency paths between entities. VERSE [22] also utilizes shortest dependency paths but uses them as features for SVM instead of neural networks, achieving the top score in the shared task. Li et al. [23] employ dynamic extended trees of given sentences as input to bidirectional LSTMs, and use SVM for post-processing. Li et al. [24] adopt an approach similar to Li et al. [23], but add attention mechanism to focus more on important information. Gupta et al. [20] also use an essentially similar neural approach based on dependency paths, except that they deal with cross-sentence events by connecting augmented paths of multiple sentences, in a way similar to graph-LSTMs of Song et al. [17]. However, they achieve the highest performance only when they set sentence range as 0 and use an ensemble of neural and non-neural linear models (i.e., detecting only intra-sentence events with the help of non-neural models). This suggests the inherent limitation of neural models trained on small data in capturing cross-sentence relations. All the existing systems employ supervised learning frameworks and optimize machine learning parameters on the BB-event training and development data.

**Table 3:** Comparison of event extraction performance

Existing models				F1	Recall	Precision
BB-event task participants (2016)	LIMSI			48.5	64.6	38.8
	TurkuNLP			52.1	44.8	**62.3**
	VERSE			55.8	61.5	51.0
State-of-the-art systems	Li et al. [23]			58.1	58.0	56.3
	Li et al. [24]			57.4	56.8	59.4
	Gupta et al. [20]			58.7	65.7	53.0
Proposed models		#intra	#cross	F1	Recall	Precision
M1: Intra-clause syntactic patterns		213	0	37.7	30.7	48.8
M2: Intra-clause syntactic patterns + trigger-based inference (train)		246	0	40.4	34.8	48.1
M3: Intra-clause syntactic patterns + trigger-based inference (unlabeled)		417	64	56.7	**68.6**	48.2
M4: VERSE (2016) + trigger-based inference (unlabeled)		339	54	**58.9**	63.8	54.9
M5: VERSE (2016) + trigger-based inference (unlabeled) (without linguistic modality detection)		339	63	58.3	63.8	53.6

The numbers in boldface indicate the highest scores for each metric

The experimental results show the practical impact of our proposed models. First of all, our purely unsupervised model achieves an F1-score of 56.7% (M3), outperforming all the supervised models presented by the shared task participants, and is compared favorably with state-of-the-art neural models. This implies that it is quite effective to use a two-step approach, where syntactically close intra-sentence relations are extracted first by explicit syntactic patterns, and then cross-sentence relations by inference on top of intra-sentence extraction. Another finding is that the use of unlabeled data to collect more diverse triggers brings considerable benefits, giving a significantly better F1-score than using only the limited training data (i.e., M3 vs. M2) through a substantial gain in recall (34.8% →68.6%). The table shows that limited types of triggers lead to a very small number of predictions with no cross-sentence event extracted. This suggests that leveraging unlabeled data on a large scale with appropriate syntactic constraints can be potentially effective at addressing the data sparsity and low coverage issues in fine-grained event extraction.

Our model is also combined with the VERSE system to produce the best performance of F1=58.9% (M4) with fewer but more precise predictions, outperforming all the three state-of-the-art models. This suggests that our trigger-based inference can be used not only to achieve reasonable performance in extracting cross-sentence events in isolation, but also to significantly benefit existing systems by allowing them to deal additionally with cross-sentence events. Moreover, since our model can be easily pipelined with any intra-sentence extraction models as seen with VERSE, we believe that it is possible to achieve even better results if our model is combined with other state-of-the-art systems such as [20].

It is also shown that even a simple approach to linguistic modality detection contributes to the overall performance, with a 1.3%p gain in precision over the modality-unaware model (M5), without any loss of recall. This suggests that it is necessary to carefully investigate and model modality-related linguistic phenomena to improve fine-grained event extraction, especially when only minimal training data is available. We believe that encoding modality information as additional features would be potentially beneficial to existing supervised systems. More sophisticated methods than simple keyword-based detection would also further improve the performance [29].

## Discussion

The experimental results demonstrate the potential of our linguistically motivated and unsupervised inference models which perform comparably with the state-of-the-art models, without any supervised learning methods. However, we still recognize a variety of challenges that are not fully addressed by our approach. In this section, we discuss them in detail with various illustrative examples to provide useful insights for future research. We also describe a multi-pass sieve architecture which partly motivates our approach, and present the intuition behind its use. The overall architecture of our system and specific approaches to extraction of BB-events are described in the “Methods” section.

### Multi-pass sieve architecture

The underlying structure of our system can be seen as a multi-pass sieve architecture, which employs a pipeline of several smaller models, or sieves. In this architecture, each of the sieves, which is designed to capture specific types of information, is applied one by one to a given document in decreasing order of precision, where earlier sieves inform later ones by transferring precise information. It was first proposed for coreference resolution [30] and then applied to other tasks such as temporal relations and quote attribution [31, 32]. It has also been employed for various tasks in the biomedical domains, such as entity linking, coreference resolution, relation extraction, and concept normalization [33–36]. Our system can be seen as consisting of four sieves that capture different types of information: (1) context triggers, (2) short-range intra-sentence (i.e., intra-clause) events, (3) long-range intra-sentence (i.e., cross-clause) events, and (4) cross-sentence events. We first extract intra-clause or short-range intra-sentence events as they involve syntactically close entities whose relationship is exhibited more clearly than syntactically distant ones, i.e., long-range intra-sentence or cross-sentence events. The extracted events in earlier sieves are then used to select candidate entities for less explicit events, which affect actual inference in the next steps (sieves). We note that this multi-pass process is essentially similar to how humans recognize information cross-sententially in a given document: They usually understand the relationship between concepts that spread across sentences, by collecting pieces of information within each sentence and using them as an underlying context. We believe that, as in the sieve architecture, it would be necessary to divide a task into several subtasks and to address each of them with specific approaches. This is expected to be particularly effective when some of the subtasks involve markedly different linguistic phenomena with minimal training data, such as cross-sentence event extraction in the BB-event task. It should be noted that most previous work on BB-event attempting to deal with cross-sentence events in a similar way to intra-sentence extraction, for example by concatenating dependency paths of multiple sentences into one larger path, has failed to extract them successfully enough to improve the overall performance [20, 22]. We also believe that it is possible to take advantage of several useful features of this architecture to further improve our approach. For example, one can quickly test ideas by implementing them as individual sieves and can add, replace and re-order them properly. This means that it is also easy to assess the contribution of each sieve to the overall performance. This is particularly useful when supervised learning does not work well, as discussed in the section on related work [30–36].

**Task-specific constraints and knowledge** As presented in the “Methods” section, we propagate event relations of bacteria-location pairs to other pairs through syntactic relationships of the two pairs between bacteria mentions or between location mentions. For example, if bacteria-location pair B-L1 is identified as an event, and at the same time L1 has a coordinate relation with another location L2 (e.g., “L1*and*L2”), we also consider the B-L2 pair as an event. Although this method works generally well in our experiments, it also produces a number of false positives in some cases, especially in the case of the *nesting* and *participle-preposition* patterns (see Table 6). We note that some of the errors can be explained by the “relation transitivity” constraints in the BB annotation guidelines, which describe for which pair of locations an event relation is transitive or not. For example, the guidelines state that if location L1 exists inside *geographical* location L2 such as “*sheep and goats in Europe*”, then any event relation that involves L1 also involves L2. By contrast, the transitivity relation does not hold between a *living organism* and an *environment of the living organism*. We found that the *patient* entities are frequently involved in this case, as shown in Example 2. In this example, each of the two sentences appearing in the same document contains two location mentions that are connected via our propagation pattern (“*LOCATION in LOCATION*”), but transitivity does not hold between them. For example, non-transitivity between L1 and L2 in the first sentence suggests that even if the bacteria strain (B1) lives in patients (L1), it does not necessarily live in the facilities accommodating them (L2). Since our system does not recognize specific types of location such as living organisms and environments, it cannot address task-specific transitivity of this kind, and always produces false positives. Example 2. Location types and relation transitivity (reference events: B1-L1, B2-L3) [PMID: 10738994]

- *… for typing methicillin-resistant* [*Staphylococcus aureus*] **B1***strains colonizing*[*patients*] **L1***in a*[*nursing home*] **L2**.
- *Prospective screening culture surveillance for* [*MRSA*] **B2***among* [*patients*] **L3***in a* [*community SNF*] **L4**.

We found that some cases are not clearly explained by the constraints stated in the annotation guidelines. In Example 3, while the transitivity relation holds between *egg products* and *egg* (i.e., L4 and L3), it does not hold between *egg pulp* and *egg* (i.e., L2 and L1). Example 3. Location types and relation transitivity (reference events: B1-L2, B2-L3, B2-L4) [PMID: 1016123]

- *Of the 104 isolations of* [*Salmonella*] **B1***sp. from* [[*egg*] **L1***pulp*] **L2**, …
- *… and subsequent detection of* [*salmonellae*] **B2***in* [[*egg*] **L3***products*] **L4**; *however, …*

It is challenging to recognize this subtle difference of semantic relations between pairs of entities, especially when they form exactly the same syntactic structure such as *egg pulp* and *egg products*. We believe that this is where supervised learning could work better, as it can automatically learn whether given mention pairs are considered positive or not, without having to understand its underlying semantics if there are sufficient training examples. We leave it as future work.

### Highly implicit events

Some cross-sentence events are highly implicitly described and involve no context triggers. They usually require multiple steps of inference and are thus difficult to extract with single pair-wise comparisons between entities. Example 4 demonstrates this case. There are one bacteria mention and two location mentions, where only one pair B1-L2 is a valid event. Our system produced two errors in this example: It incorrectly identified the B1-L1 pair as an event (i.e., false positive) by using *disease* (underlined) as a trigger for B1, and missed the B1-L2 pair (i.e., false negative) because there is no context trigger associated with L2. In order to detect the event correctly in this case, it is necessary to perform a chain of logical inference, such as how bacteria strains affect the gene expression of particular DNAs, and how it is related to the morphogenesis of the organism. It is also challenging in this example to distinguish a valid cross-sentence event (B1-L2) from an invalid one (B1-L1) expressed in the same sentence. Example 4. Highly implicit cross-sentence event (reference event: B1-L2) [PMID: 9526514]

- [*Agrobacterium rhizogenes*] **B1***strains of the agropine type harbor on their Ri-plasmid two T-DNAs, a left TL-DNA and a right TR-DNA.*
- *The rolB gene of the TL-DNA is the major factor in the pathogenesis of the* [*hairy-root*] **L1***disease and its constitutive expression interfere profoundly with* [*plant*] **L2***morphogenesis.*

Highly implicit events of this kind are also found within a sentence. Example 5 shows two intra-sentence events involving syntactically distant bacteria-location pairs whose relationship is implicitly described. For the first cross-clause event B1-L1, it is necessary to find that *MRSA-contaminated surfaces* are part of *correctional facilities* (i.e., a meronym relation), which is not clearly evidenced through any syntactic information in this sentence. For the second event B2-L1, words such as *cleaning* and *prevention* suggest the existence of the *MRSA* bacteria in the *facilities*. Although our system recognized two relevant context triggers *contaminated* and *prevention*, it failed to detect both the two events since the triggers are not connected to L1 via our syntactic rules. This example highlights the necessity of exploiting word-level semantics, especially for the relationship between location entities. Example 5. Highly implicit intra-sentence events (reference events: B1-L1, B2-L1) [PMID: 19622846]

- *Finding* [*MRSA*] **B1***-contaminated surfaces on a variety of environmental surfaces in the absence of an overt outbreak emphasizes that* [*correctional facilities*] **L1***should have protocols for environmental cleaning as a component of* [*MRSA*] **B2***prevention.*

### Linguistic modalities

Although we use the syntactic relationship between modality keywords and entity mentions (or triggers), some modalities are too implicitly expressed to be detected by a few keywords, resulting in a number of false positives.

Example 6 shows two consecutive sentences found in the training data containing two bacteria mentions B1 and B2 and three location mentions L1, L2, and L3, where L1 has an event relation with B1 and B2 through an intra-clause syntactic pattern whereas both L2 and L3 do not have an event relation with any bacteria mention. Example 6. False positives in the hypothetical statement (reference events: B1-L1 and B2-L1) [PMID: 25098305]

- *Collectively, these data indicate that both* [*M. agassizii*] **B1***and* [*M. testudineum*] **B2***are present in* [*Georgia populations of gopher tortoises*] **L1***and that clinical disease is apparent in populations where both pathogens are present.*
- *Additional research is needed to better understand the role of these twopathogens, and other potential pathogens, in the overall health of* [*tortoise populations*] **L2***, especially if future conservation efforts involve translocation of*[*tortoises*] **L3**.

Here, our system correctly identified two intra-sentence events B1-L1 and B2-L1, but incorrectly identified two (false-positive) cross-sentence events B1-L2 and B2-L2. This is because our system treated *pathogens* (underlined) in the second sentence as a context trigger for both B1 and B2 in the first sentence (which indeed acts as their anaphora), and associated it with L2 via an intra-sentence syntactic pattern. However, the second sentence does not confirm the relationship between the *two pathogens* and the *tortoise populations*, but just emphasizes the necessity of investigating it in the future, as indicated by “*needed to better understand*”. It is thus difficult for our system to capture implicitly expressed modalities of this kind with just a few keywords. For L3, our system correctly excluded it from an event (i.e., true-negative) as it is contained in the if-clause.

Another important implication of this example is that such linguistic modalities are usually signaled by keywords or phrases outside the shortest dependency path between given entities. This means that information obtained from the shortest dependency path might not be sufficient enough to distinguish between various linguistic modalities. For example, the shortest dependency path from *pathogens* to *tortoise populations* (i.e., “*pathogens* →*of* →*role* →*in* →*health* →*populations*”) does not include any word of “*needed to better understand*” which is key to modality detection in this example. This could be an important issue because most of the recent supervised systems rely only on the information available on the shortest dependency path, discarding all the information outside it in order to improve generalization. We also encounter this issue in extracting intra-sentence events as shown in Example 7. Example 7. False positive in the research goal statement (no reference event) [PMID: 20073421]

- *To evaluate the growth potential of*[*F. tularensis LVS*] **B1***strain in* [*macrophage-like cell line J774*] **L1***modulated by …*

Here, although B1 is syntactically close to L1, they do not form an event. This is because the sentence just describes a research goal, and does not present any evidence or confirmation for their relationship, as indicated by the phrase “*To evaluate*” in the beginning of the sentence. However, this phrase is accessible only outside the shortest dependency path between B1 and L1, suggesting that it is necessary to check all the information in the sentence, even when extracting intra-sentence events. Our system correctly classified this case as a non-event (i.e., true-negative) using the keyword *evaluate* and its syntactic relationship with B1 and L1.

Aside from these hypothesis and research goal statements, there are also various forms of negation that affect the decision on event relations. Example 8 shows three sentences containing negated expressions and single bacteria-location pairs. While the first two sentences contain no event annotation, the third still contains a valid event relation despite the explicitly expressed negation. Example 8. Three different forms of negation that affect event relations [PMID: 10658649, 2696427, 8532424]

- *The interaction between* [*Streptococcus pyogenes*] **BACTERIA***and the* [*host cell*] **LOCATION***surface is ****not**** completely understood.*
- ***No*** [*V. salmonicida*] **BACTERIA***could be detected in* [*sediments*] **LOCATION** …
- ***None of**** the* [*colonized children*] **LOCATION***experienced an invasive* [*K. kingae*] **BACTERIA***infection.*

Note that all the three sentences exhibit different levels of difficulties in determining how the negation affects event relations. In the first sentence, while the negation must be considered for correctly classifying the pair as a non-event, it can be captured only outside the shortest dependency path between the two entities. The second sentence is a rather straightforward case: The use of the determiner *No*, which directly modifies the bacteria mention, indicates that no event relation holds between the pair. By contrast, the third one requires a more careful analysis: Even though the negation expression *None of* modifies the location mention in a similar way to the second sentence, the pair is still annotated as a valid event in the training data. This is because the sentence means that the event relation does not hold just for the “*invasive*” *K. kingae bacteria*, but is still valid for general types of the *K. kingae bacteria*. Our system does not perform such sophisticated logical analysis, producing a false-positive result for this sentence.

Some bacteria-location pairs are also expressed with a loose causal relationship, making it difficult to judge how strongly they are related, even by humans. In Example 9, our system made erroneous decisions by identifying B1-L1 as an event through their syntactic relationship, and propagating it to L2 via the participle-preposition pattern (i.e., *known as*) and to L3 via the apposition pattern. This produces a cascade of errors. The fact that this example has no reference event annotation implies that even if those who are infected with bacteria have “a history of consumption” of organisms, it does not necessarily mean the existence of the bacteria in the organisms. A more careful analysis would be needed to deal with such a loosely established causal relationship. Example 9. Unconfirmed causal relationship between bacteria and locations (no reference event) [PMID: 6107735]

- *All but 1 of the 12 people with* [*V. cholerae O:1*] **B1***infection ****gave a history of recent consumption**** of* [*marine bivalves*] **L1***known locally as* [*arselle*] **L2** ([*pelecypods*] **L3**).

### Coreference and anaphora resolution

In order to use context triggers as indirect anaphors of bacteria mentions, our system relies on a simple strategy, associating each trigger to the bacteria mention closest to it. We found that this generally works well, but in some cases it is necessary to apply a more sophisticated mapping, especially based on coreference or anaphora resolution. Example 10 shows how anaphora resolution can be potentially useful in creating a correct mapping. Example 10. Anaphora resolution for cross-sentence inference (reference event: B1-L2) [PMID: 8347510]

- *Although* [*A. aquaeolei*] **B1***is most closely related to* [*purple sulfur bacteria*] **B2***(the genera* [*Ectothiorhodospira*] **B3***and* [*Chromatium*] **B4**)*, it is not a* [*phototrophic microorganism*] **L1***, which is consistent with ****its****isolation from a* [*subterranean environment*] **L2**.

Here, our system recognizes *isolation* (underlined) as a context trigger, and associates it with the closest bacteria mention B4. Since this trigger is connected to L2 via one of our intra-clause syntactic rules, our system considered the B4-L2 pair as an event by transitivity, making an erroneous decision (i.e., false-positive). The correct bacteria mention that must be mapped to *isolation* is B1, and the B1-L2 pair is the only correct event in this example. Here, anaphora resolution can be helpful as it is used to determine which bacteria mention is involved in *isolation*, by identifying the antecedent of the possessive pronoun *its*. Although the BB-event annotations contain coreference relations, they are limited to only a few explicit cases such as appositives and abbreviations, and do not cover pronouns at all, as seen in the previous edition of the BB-event tasks [37].

Example 11 also shows the necessity of domain-specific coreference resolution to correctly associate context triggers to bacteria mentions. Example 11. Coreference resolution for cross-sentence inference (reference events: B1-L1, B1-L2) [PMID: 8347510]

- *We present the real-time monitoring of hydrogen cyanide (HCN) production from* [*Pseudomonas aeruginosa*] **B1***(P. aeruginosa) strains in vitro, using laser-based photoacoustic spectroscopy.*
- *Both reference strains and* [*clinical*] **L1***isolates of* [*patients with CF*] **L2***were studied, and compared to ****other****pathogens commonly present in* [*lungs/airways of CF patients*] **L3**.

The two sentences in this example appear in a single document and contain two cross-sentence events B1-L1 and B1-L2. Our system correctly identified these two events by associating the trigger *isolates* (underlined in the second sentence) with B1 (which is closest to *isolates*) and by finding the intra-sentence relations the trigger has with L1 and L2. However, our system incorrectly identified (i.e., as false positive) B1-L3 as an event because it considered *pathogens* (underlined in the second sentence) as a trigger for B1 (which is closest to *pathogens*) and linked it to L3 via an intra-sentence rule. Here, the modifier *other* suggests that *pathogens* do not refer to any specific bacteria mentioned in the example. Hence, it must not be used as a medium for inference. We believe that it is necessary and interesting future work to study how biomedical coreference resolution can be used to establish a correct mapping between context triggers and bacteria mentions and how it benefits implicit event extraction.

### Generic vs. specific entities

We note that the level of specificity of entities affects how they are related with other entities. For example, we found that generic entities tend not to have an event relation with specific entities, as shown in Example 12. Example 12. Different levels of specificity of entities (reference events: B1-L1, B2-L4, B2-L2) [PMID: 10738994]

- *Prospective screening culture surveillance for* [*MRSA*] **B1***among* [*patients*] **L1***in a community SNF …*
- [*Nares and stool swab cultures*] **L2***were obtained from newly admitted* [*patients*] **L3** …
- [*MRSA*] **B2***were isolated by* [*oxacillin screening agar*] **L4**.

The example shows three consecutive sentences found in a single document from the training data, containing annotations of three events: two intra-sentence events B1-L1 and B2-L4, and one cross-sentence event B2-L2. However, no event relation holds for the B1-L2 pair, despite exactly the same surface form of B1 and B2 (i.e., *MRSA*). The difference comes from the specificity of entities: B1 refers to a generic type of entity, whereas B2 and L2 more specific ones. This specificity is exhibited on a sentence level. The second and third sentences together describe a single specific experimental process, and hence the annotated mentions in these two sentences also refer to entities realized in a specific environment. By contrast, the first sentence is just used to introduce an experimental design prior to the specific experimental process and result, which explains why B1 does not have any event relation with the entities in the second and third sentences. This example highlights the necessity of identifying this subtle difference between entities, especially for fine-grained event extraction.

**Fig. 1:**
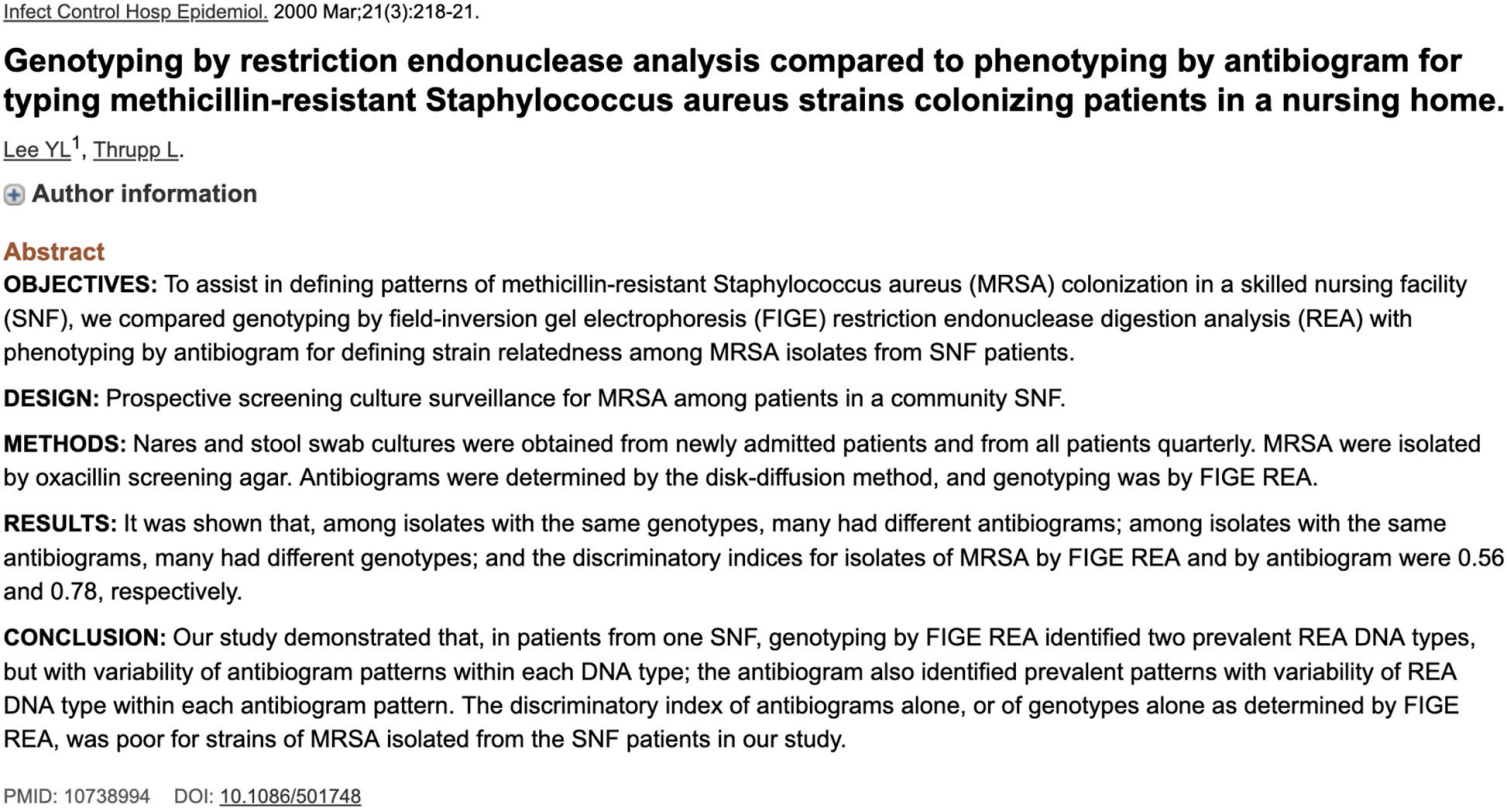
Screenshot of the PubMed original abstract text for the BB-event document (PMID: 10738994). The BB-event datasets do not contain headers such as OBJECTIVES, DESIGN, and METHODS

While it is challenging to determine the degree of specificity of this kind, we notice that ’paragraph headers’ can be useful information for this; our analysis of the original articles of the BB-event datasets reveals that a single biomedical abstract is usually divided into several segments (or paragraphs) where each of them starts with headers that indicate for what purpose it is described, such as *Design*, *Methods*, and *Results*. These headers are good indicators of whether a given sentence describes specific experiments/results or not. However, they are missing in the official BB-event data, even when they are found in the original articles. Figure 1 shows the original abstract text for Example 12 found in the PubMed website, indicating that the first sentence in Example 12 corresponds to *Design*, whereas the second and third sentences Methods.

### Applicability to other types of events and relations

Our method for context trigger identification has the potential to deal with a variety of events and relations across tasks. More specifically, we can use intra-sentence extraction rules to collect context triggers for other types of events and relations. For example, in the case of chemical-disease relations in the BioCreative V Chemical Disease Relation (CDR) Task [8], one of the syntactic patterns that strongly signal valid relations would be “{noun} of CHEMICAL {verbs} DISEASE”. In this pattern, expressions matched by “{noun}” would be considered as a context trigger, in a way similar to our proposed method for the BB-event task (Table 4). In order to examine the feasibility of this idea, we performed a pilot study, where we collected sentences from PubMed and PMC using this syntactic pattern together with the ten most frequent chemical entities taken from the CDR training data, such as *cocaine*, *dopamine*, *morphine*, and *nicotine*. We also used three verbs generally indicative of causal relationship for the trigger pattern: *cause*, *lead*, and *show*. We then filtered out sentences that do not mention any of the disease entities in the CDR training data. As a result, we were able to collect from the remaining sentences several promising disease-related context triggers for chemical entities such as *administration*, *injection*, *concentration*, *ingestion*, *toxicity*, *discontinuation*, *withdrawal*, and *cessation*. The following examples show four collected sentences sharing the same chemical entity *morphine* but different potential context triggers (underlined).

**Table 4:** Syntactic patterns for collecting context triggers

	Trigger patterns	Examples
1	{*noun*} *of* [*bacteria*] *loc_prep* [*location*]	∙*elimination of* [*Helicobacter pylori*] **B***in the* [*antrum*] **L**
		∙*attachment of* [*Escherichia coli O157:H7*] **B***to* [*apple surfaces*] **L**
2	[*bacteria*] {*n/v/p*} *loc_prep*[*location*]	∙*Heat-killed* [*C. burnetii*] **B***purified with* [*normal yolk sacs*] **L**
		∙ [*Non-O1 Vibrio cholerae*] **B***bacteremia in* [*patients with cirrhosis*] **L**
3	[*location*] {n/v/p} *prep* [*bacteria*]	∙ [*Respiratory*] **L**carriage* of* [*Kingella kingae*] **B**
		∙*Twenty-eight* [*blood culture*] **L**isolates* of* [*non-O1 V. cholera*] **B**

Underlined expressions are context triggers to be collected. [*bacteria*] and [*location*] are bacteria and location mentions annotated in BB-event training data, respectively. *prep* and *loc_prep* are a general preposition and a locational preposition, respectively. *{n/v/p}* is either a noun, a verb, or a participle. Examples on the right show actual snippets from the BB-event training data matched by syntactic patterns on the left. B and L in boldface refer to bacteria and location entities, respectively

Example 13. Sentences collected from PubMed and PMC using the trigger patterns and chemical entities

- *we have now shown that equimolar concentrations of* [*morphine*] **CHEMICAL***, methadone, and buprenorphine show similar,* [*neurotoxic*] **DISEASE***interactions with Tat.* [PMCID: PMC4475822]
- *the intravenous (IV) acute injection of* [*morphine*] **CHEMICAL***in rabbits caused* [*hypertension*] **DISEASE***, bradycardia, and hyperglycemia.* [PMCID: PMC3347855]
- *intrathecal administration of sufentanil, fentanyl, and* [*morphine*] **CHEMICAL***to dogs led to no histological signs of* [*neurotoxicity*] **DISEASE***after 28 days of daily exposure.* [PMCID: PMC4565585]
- *Abrupt cessation of* [*morphine*] **CHEMICAL***leads to withdrawal signs and* [*cognitive deficits*] **DISEASE**. [PMID: 24459477]

These context triggers can be directly used for cross-sentence relation extraction in the CDR task. For instance, the following example of two consecutive sentences taken from the CDR abstract text shows that the cross-sentence relation between *Midazolam hydrochloride* (chemical) and *respiratory and cardiovascular depression* (disease) can be inferred via the context trigger *administration* (underlined in the second sentence) as this trigger naturally implies the medical use of *Midazolam hydrochloride* mentioned in the first sentence. Example 14. Use of a context trigger for cross-sentence chemical-disease relations [PMID: 2375138]

- [*Midazolam hydrochloride*] **CHEMICAL***is commonly used for dental or endoscopic procedures.*
- *Although generally consisted safe when given intramuscularly, intravenousadministration is known to cause* [*respiratory and cardiovascular depression*] **DISEASE**.

Note that this process is very similar to how we collect and use context triggers to extract cross-sentence bacteria-location relations. Although a more detailed analysis would be needed for future work, this example suggests that our context trigger identification is broadly applicable to other types of event/relation extraction.

## Conclusions

In this paper, we address implicit event extraction for the bacteria-location relationships, with a particular focus on cross-sentence events that most existing work has treated as false positives due to limited training examples. We adopt an unsupervised approach based on context triggers. They signal the presence of bacteria entities that are likely to be mentioned in adjacent sentences in a way similar to anaphora, and thus provide important information for cross-sentence inference. We propose a method for collecting salient and diverse trigger expressions from large-scale unlabeled text using linguistically motivated syntactic constraints, without any external knowledge and curated databases. Our trigger-based inference model significantly outperforms all the shared task participants, and is compared favorably with state-of-the-art neural models when combined with the existing supervised intra-sentence extraction system. We expect that our method can be used to achieve further improvement by combining with more advanced models for intra-sentence extraction and by using context triggers as features for supervised learning of cross-sentence events. We also believe that it would be interesting future work to investigate how well our method for collecting context triggers is applicable to other types of cross-sentence events or relations across various domains.

## Methods

### Overview

**Fig. 2:**
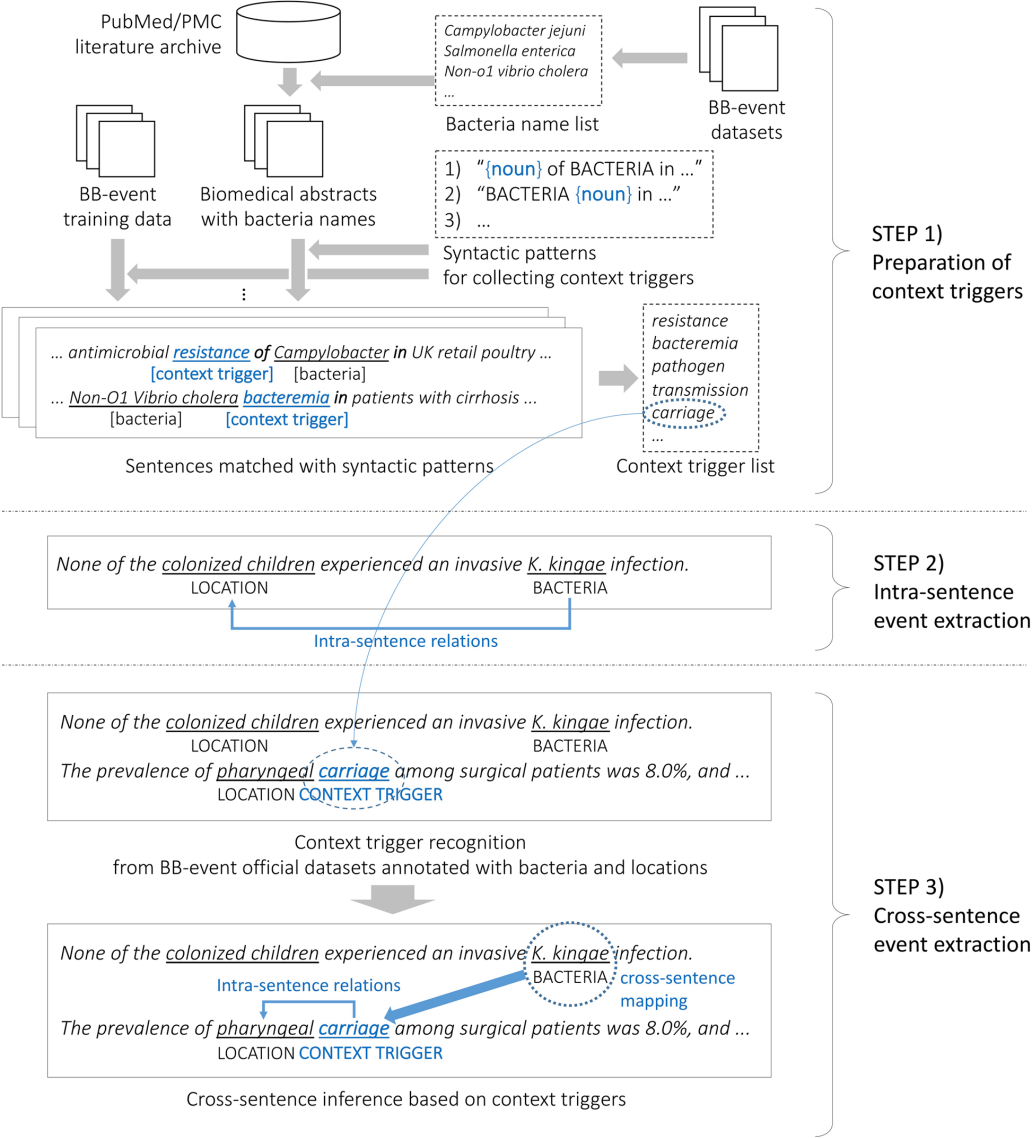
Overview of the proposed model

The overall workflow of the proposed approach is illustrated in Fig. 2. It consists of three steps, as follows. First, we collect context triggers to be used for cross-sentence inference, from large-scale plain texts. For this process, we use a list of bacteria names compiled from official BB-event annotated datasets and linguistically motivated syntactic patterns to be compared against the output of a dependency parser. Second, we extract intra-sentence events, where bacteria and location entities are mentioned in the same sentence (*K. kingae* →*colonized children*), using either syntactic rules or existing intra-sentence event extraction systems. Third, we detect all the occurrences of context triggers from a given BB-event document (where events are to be extracted) using the list of triggers compiled in the first step (e.g., *carriage*). We then create the mapping from bacteria mentions into the context triggers in adjacent sentences (e.g., *K. kingae* →*carriage*), and infer cross-sentence events by detecting intra-sentence relations between context triggers and location mentions (e.g., *carriage* →*pharyngeal*). Note that creating a list of context triggers from unlabeled texts is performed only once (the first step), and the same list is re-used for event extraction across all the BB-event documents (the second and third steps). In the remaining part of this section, we describe each part and our intuition behind it in detail.

### Collecting context triggers

**Fig. 3:**
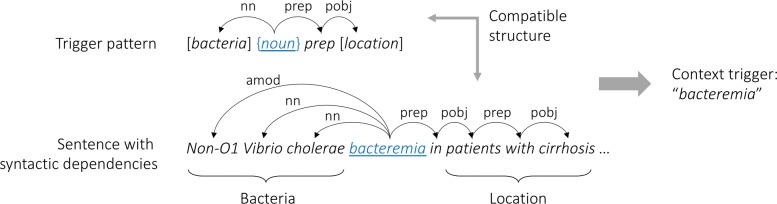
Using a trigger pattern to collect a context trigger from training data

As described in the “Background” section, the key idea of our approach is to use context triggers for cross-sentence inference that act as an indirect anaphor for bacteria entities mentioned around them. Our intuition behind collecting such triggers is two-fold. First, certain types of syntactic constructions provide information about how bacteria are involved in a location. Second, these constructions are likely to contain expressions that play an important role in triggering such spatial context of bacteria. More important, these expressions frequently appear without bacteria mentions in a sentence but still imply the presence of bacteria, making it possible to use them for cross-sentence inference. We use three types of linguistically motivated syntactic patterns that trigger spatial context of bacteria as shown in Table 4, with blue underlined expressions to be collected as context triggers. Each pattern is paired with its underlying syntactic dependency structure, as exemplified in Fig. 3.

In Table 4, the examples on the right show that the recognized (underlined and in blue) context triggers such as *elimination* and *attachment* refer to a bacteria-related process involving a physical location. Figure 3 also shows an example of collecting context triggers using these trigger patterns; for every possible intra-sentence pair of bacteria and location mentions annotated in the training data (e.g., *Non-O1 Vibrio cholerae* and *patients with cirrhosis* in Fig. 3), we first process the corresponding sentence using the Stanford dependency parser [38] to obtain its syntactic dependency structure. We then check if the syntactic dependency between the bacteria-location pair is compatible with one of the trigger patterns in Table 4. If so, we identify and collect a context trigger from the corresponding matched pattern (e.g., *bacteremia* in Fig. 3). For *loc_prep* in the first two patterns, we use only locational prepositions such as *in*, *on*, *to*, *from*, and *at* in order to further limit the semantics of [*location*] to actual locational entities for bacteria, and all prepositions for the last pattern.

One limitation of collecting context triggers in the way above is that only a few sentences are matched by the trigger patterns due to the limited size of training data, ending up with only a small number of triggers collected. They would not be sufficient enough to generalize to unseen data, given that the test data is larger than the training data in the BB-event task (e.g., 327 and 340 events in the training and test data, respectively). We address this issue by applying the trigger patterns to a large amount of unlabeled plain text to collect much more diverse context triggers. The problem is that this may result in collecting noisy and useless expressions due to the lack of bacteria and location annotations in plain text that help to select candidate sentences to be matched with the trigger patterns. We note, however, that the number of unique bacteria expressions found in the literature is usually limited and that our purpose is to extract context triggers from sentences containing bacteria mentions, but not to extract bacteria mentions themselves. We thus chose to use a pre-defined set of bacteria names rather than trying to recognize unseen names from plain text. For this purpose, we compiled a list of bacteria names by collecting all the bacteria mentions annotated in the official BB-event training, development, and test datasets, obtaining 317 distinct bacteria names. We then used them to select initial candidate sentences from plain text. For location expressions, we did not use this type of pre-compiled list and allowed any expressions to be positioned at [*location*] in the patterns. This is based on the assumption that locational prepositions such as *in* and *from* can properly control the semantics of those expressions.

As shown in Fig. 2, we used the PubMed and PMC search engines to extract sentences from biomedical literature abstracts and full-text articles that contain at least one of the bacteria names in our compiled list and at least one preposition. We use the longest match as some bacteria names are nested in others, such as *campylobacter* and *campylobacter jejuni*. Once sentences mentioning bacteria are identified from plain text, we compared them with the trigger patterns and collected matched trigger expressions in the same way as in Fig. 3. Here, we did not try to match the [*location*] slot in the trigger patterns, as explained above. Expressions whose frequency is lower than empirical thresholds (to be detailed in the “Results” section) are excluded. All the remaining triggers are lemmatized and nominalized by the WordNet lemmatizer available in the Natural Language Toolkit [39].

Although the proposed patterns can be used to collect context triggers from plain text, they also result in a number of expressions that are unlikely to act as context triggers. This is because these patterns actually cover a wide range of expressions irrelevant to specific biomedical concepts, and because the syntactic parser sometimes produces incorrect dependency graphs, matching many unintended ones. Hence, we manually analyzed and filtered out such non-trigger expressions to enable precise inference. Our analysis revealed that there are especially two types, among others, of such expressions as shown below.

1. Research purpose or methods, such as *analysis*, *study*, *examination*, *evaluation*, *inspection*, *assessment*, and *investigation*
  e.g., *An evaluation of*[*selectivebroths*] **BACTERIA***based on the bi-selenite ion and on hypertonic strontium chloride in* [*Salmonellae*] **LOCATION***detection*
2. A certain amount, change of the amount, or class of bacteria, such as *any*, *some*, *part*, *number*, *amount*, *frequency*, *type*, *pattern*, *class*, *group*, *range*, *change*, *increase*, and *decrease*
  e.g., *A range of* [*Chlamydia trachomatis*] **BACTERIA***strains in* [*cycloheximide-treated McCoy cells*] **LOCATION**

Note that there could be other types of non-trigger expressions and that there is still much room for improvement on how to filter them out. More sophisticated and automated tools can be adopted for this. One example is information retrieval techniques [40, 41] that assess how likely they are to occur with bacteria and location mentions in text. Another example is domain-specific continuous representations of words (i.e., word vectors) [42, 43] that determine their distributional relevance to biomedical entities and processes. We leave this as future work. Details about the collected context triggers are presented in the “Results” section.

Once the list of context triggers is compiled through the process above, we can identify all the occurrences of context triggers appearing in the given BB-event document from which events are to be extracted, such as *carriage* in Fig. 2, and then use them for cross-sentence inference. We consider all the nouns, verbs, and adjectives as candidate triggers and compare them with the ones in the trigger list based on stemming, so that verb and adjective triggers can also be matched with noun triggers. For example, if the verb *persist* appears in a given document, we treat it as a valid trigger because it matches with *persistence* in our trigger list via stemming-based comparison. We used the Porter stemmer available in the Natural Language Toolkit [39], by which we could unambiguously identify all the valid verb/adjective triggers.

### Intra-sentence event extraction

We use two approaches to intra-sentence event extraction : (1) the use of systems proposed by other researchers and (2) our rule-based extraction with context triggers. The first approach is motivated by the availability of supervised systems for extracting intra-sentence events. Since our method for cross-sentence extraction can be easily pipelined with such systems, we first extract as many intra-sentence events as possible using such an existing system, and then extract cross-sentence events from the remaining candidate bacteria-location pairs using our trigger-based inference. We use the VERSE system [22] as it is the only publicly available one at the time of writing. It is a supervised binary classification system trained with SVM and linguistic features such as surface tokens, part-of-speech tags, and syntactic dependency paths. It achieved the highest F1-score in the BB-event shared task.

The second rule-based approach is motivated by the possibility of using context triggers to detect long-range intra-sentence events in a way similar to cross-sentence inference. Our intuition behind it is that both long-range intra-sentence and cross-sentence events pose essentially the same challenge; while most state-of-the-art systems consider shortest dependency paths between involved entities as a prerequisite for generating underlying text representations [20, 23, 24, 28], such syntactic dependencies are less effective at modeling long-distance entity pairs in text. We test how well our unsupervised trigger-based inference extracts them by comparing it with state-of-the-art supervised models. The remainder of this section is focused on describing our trigger-based approach to both long-range intra-sentence and cross-sentence events.

We divide intra-sentence events into two classes: intra-clause (short-range) events and cross-clause (long-range) events. Note that cross-clause events involve bacteria and location entities that are positioned in different clauses, and that are thus likely to be distant from each other with respect to syntactic dependencies.

**Intra-clause event extraction** We first extract only the syntactically close (i.e., intra-clause) bacteria-location pairs using linguistically motivated syntactic rules that intuitively signal the presence of an event. We use two sets of rules. First, we re-use all the trigger patterns in Table 4, as they also cover syntactically close valid relations between bacteria and locations. If a candidate bacteria-location pair is matched with one of the trigger patterns, we consider it as a valid intra-clause event. Second, we also employ additional three syntactic rules to extract more tightly connected intra-clause events as shown in Table 5. Our analysis showed that such syntactically close relations capture a strong semantic association between bacteria and locations, and that they can thus be used to extract intra-sentence events with a higher precision.

**Table 5:** Syntactic patterns for extracting additional intra-clause events

	Intra-clause syntactic patterns	Examples
1	{[bacteria] [location]} NP or {[location] [bacteria]} NP i.e., bacteria and location mentions are nested in a longer noun phrase	∙*Presence of the* [*fish*] **L***pathogen* [*Vibrio salmonicida*] **B**
		∙*Prevention and treatment of* [*Staphylococcus*] **B** [*biofilms*] **L**.
2	[bacteria [location]] or [location [bacteria]] i.e., a bacteria mention is nested within a location mention or vice versa.	∙ [[*Mtb*] **B***-infected human blood monocyte*] **L**
		∙*All but 1 of the 12* [*people with* [*V. cholerae O:1*] **B***infection*] **L**
3	[bacteria] prep [location] or [location] prep [bacteria]	∙*an emerging mechanism of resistance in* [*S. enterica*] **B***in the two* [*studied hospitals*] **L**

B and L in boldface refer to bacteria and location entities, respectively

**Fig. 4:**
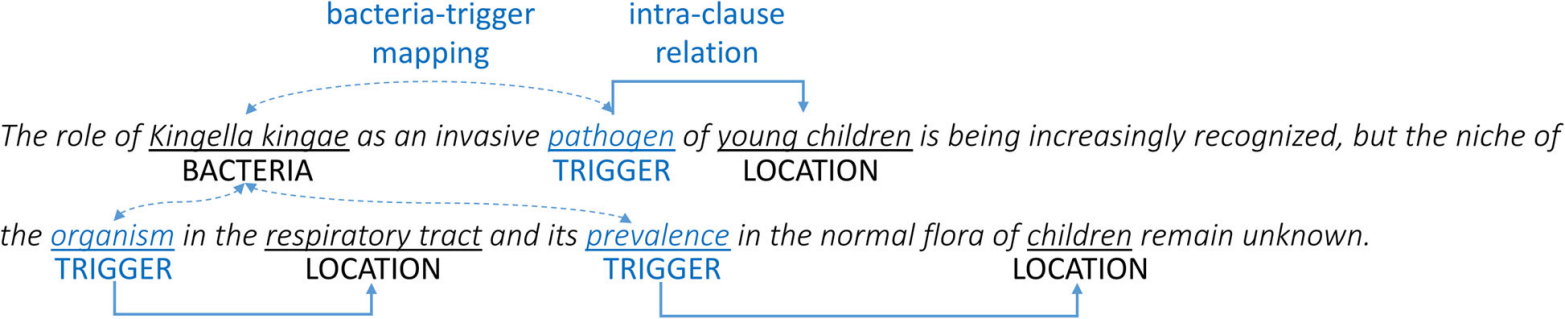
Example of extracting long-range intra-sentence events

**Cross-clause event extraction** Once intra-clause events are extracted, we infer cross-clause events using context triggers. The process is essentially similar to that for cross-sentence events: If a single sentence contains bacteria B, location L, and trigger T for B (i.e., T is already associated with B), with T and L in the same clause, we check if T can be associated with L using the syntactic rules (Table 5) to form an intra-clause event. If so, by transitivity we consider the pair of B and L as an intra-sentence event in a way similar to cross-sentence inference. Figure 4 shows how long-range (i.e., cross-clause) intra-sentence events are extracted from a single sentence, through a three-step process of trigger-based inference as follows.

1. Identify all the occurrences of context triggers: *pathogen*, *organism*, and *prevalence*.
2. Link each trigger with a bacteria mention, i.e., creating indirect coreference relations between them. In this example, as *Kingella kingae* is the only annotated bacteria mention, we link all the triggers to it (dashed arrows).
3. Extract all the possible intra-clause events using the syntactic rules (Tables 1 & 2), where each trigger is treated as a bacteria mention. In this example, there are three extracted events between triggers and locations (solid arrows): <*pathogen*, *young children*>, <*organism*, *respiratory tract*>, and <*prevalence*, *children*>.

Note that as sentences may include more than one bacteria mention, it is necessary to create an appropriate mapping between bacteria mentions and context triggers. We adopt a simple strategy for this: For each bacteria mention, we calculate how close it is to each trigger simply by counting the number of words between them and choose the closest one. This is to make sure that each bacteria mention is mapped only to one trigger closest to it. Although we found that this simple mapping works reasonably well, there would be more sophisticated strategies, such as using syntactic dependencies or linguistic rules used in anaphora resolution. We leave them as future work.

**Fig. 5:**
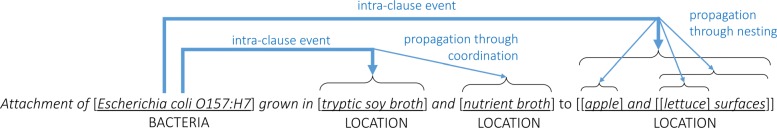
Example of propagating event labels to other pairs of bacteria and locations

**Intra-sentence event propagation** In order to compensate for the potentially low recall due to using direct syntactic dependencies of bacteria-location (or trigger-location) pairs, we employ another set of linguistically motivated syntactic rules to detect additional events based on the events extracted so far. More specifically, if we extract event B1-L1 using the proposed intra-clause rules, and at the same time another location L2 has a particular syntactic relationship with L1, we also consider the B1-L2 pair as another event. Likewise, if another bacteria B2 has such a relationship with B1, new event B2-L1 is extracted. For example, if pair B1-L1 is found to be an event, and L1 forms a coordinate clause with another location L2, such as “L1*and*L2” in a sentence, we consider the B1-L2 pair as another valid event. This can be seen as propagating event label B1-L1 to another candidate pair B1-L2. We use five types of syntactic relations between bacteria/trigger mentions or between location mentions as propagation conditions, as shown in Table 6. All these patterns are easily captured by the dependency parser. Figure 5 also illustrates how event labels are propagated through nesting and coordination. Note that these rules are applied repeatedly to all of the remaining bacteria-location (or trigger-location) pairs in each sentence wherever possible, until no more pairs are identified as an event by propagation. This repeated process is to deal with cases where multiple patterns are found in a row such as coordination of several mentions, or are embedded in other patterns such as coordination of mentions that nest another mention in them (i.e., nesting is embedded in coordination).

**Table 6:** Syntactic patterns for propagating event labels to other candidate bacteria-location (or trigger-location) pairs, with examples where B, B1 and B2 are bacteria annotations, and L1 and L2 are location annotations

	Propagation patterns	Descriptions and examples
1	Nesting	Given the phrase “*apple and lettuce surfaces*” with its four nested location annotations: *apple surfaces*, *lettuce surfaces*, *apple*, and *lettuce*, if bacteria **B** is found to be located in “*apple and lettuce surfaces*”, we identify four events by associating **B** with each of the four location mentions, as illustrated in Fig. 4.
2	Coordination	Given the two coordinated location mentions “*tryptic soy broth*” and “*nutrient broth*”, if bacteria **B** is found to be located in “*tryptic soy broth*”, we also identify the pair of **B** and “*nutrient broth*” as an event, as illustrated in Fig. 4.
3	Apposition	In the example “[*methicillin-resistant Staphylococcus aureus*] **B1***(*[*MRSA*] **B2***)colonization in a* [*skilled nursing facility*] **L1***(*[*SNF*] **L2***)*”, we first identify the intra-clause event **B1**-**L1** using a trigger pattern, and then propagate it to additional three events **B2**-**L1**, **B2**-**L2**, and **B1**-**L2**, based on the apposition relations between **B1** and **B2** and between **L1** and **L2**, as captured by their syntactic dependencies.
4	Location hierarchy	Two geographical location mentions are sometimes connected via a comma when they have a clear hierarchical relationship, such as “*in* [*Georgia*] **L1***,* [*USA*] **L2**” and “*residents of* [*Olmsted Country*] **L3**, [*Minnesota*] **L4**”. In this case, we propagate an event relation for a smaller region to that for a larger one.
5	Participle- preposition	In the example “[*Israeli travelers*]L1 *returning from* [*Nepal*]L2 *were diagnosed with* [*S. Paratyphi A*]B1 *bacteremia*”, we first identify the intra-clause event **B1**-**L1** using a trigger pattern, and then propagate it to **B1**-**L2** through the participle-preposition relation between **L1** and **L2**. This pattern can also be applied to two locations connected by a single preposition such as “*in the* [*Hospital S. Camillo De Lellis*]**L1***of* [*Roma*]**L2**”.

Note that these patterns are applied to entity pairs of the same type, i.e., bacteria-bacteria or location-location pairs. B, B1, and B2 in boldface refer to bacteria entities. L, L1, L2, L3, and L4 in boldface refer to location entities

### Cross-sentence event extraction

Extraction of cross-sentence events is essentially similar to trigger-based intra-sentence extraction: Creating the cross-sentence mapping from bacteria mentions into context triggers, and then detecting intra-sentence relations between context triggers and location mentions using linguistic rules. Aside from this, there is also another important issue: Which pairs of entities in a document are considered as candidates for cross-sentence events. This issue depends heavily on the task characteristics and thus must be addressed in a task-specific manner. In particular, we found that whether or not an entity is involved in cross-sentence events depends on whether or not it is already involved in intra-sentence events. For example, previous work on finding event locations (verb-location links) from a document [19] restricts each verb to be linked with only one (i.e., best-fit) location, whether or not the link is created within the sentence boundary. This means that if a verb already has an intra-sentence link with any location, it can never be linked with locations in other sentences. By contrast, the BB-event task allows bacteria mentions to be associated with more than one location, either intra-sententially or cross-sententially. This difference suggests that it is necessary to select candidate mentions for cross-sentence events in a task-specific manner, especially by examining how they are involved in intra-sentence events.

From our analysis of cross-sentence events annotated in the BB-event training and development data, we make the following three observations that lead to appropriate criteria for selecting candidate entity mentions to be involved in cross-sentence events:

1. If a bacteria mention has an intra-sentence relation with any location mention, it is more likely to be involved in cross-sentence events than those that have no relation with any location mention existing in the same sentence. We conjecture that this is because the literature frequently describes a situation where one bacteria is involved in several different biological processes. This involvement is annotated in the form of multiple links from one bacteria mention to multiple location mentions across more than one sentence. This is different from [19], whose goal is to find only the single most appropriate location for each event (verb).
2. If a bacteria entity is mentioned but no location mention exists in a sentence, it is likely to have an event relation with another location in adjacent sentences. We observe that this happens when a single relation between bacteria and location entities is expressed across two sentences, without any other entities mentioned between them.
3. If a location mention already has an intra-sentence relation with any bacteria mention, it never has a cross-sentence relation with any other bacteria mention. We found that two different bacteria entities mentioned in different sentences are rarely associated with one location mention at the same time, especially if one of them lies in the same sentence as the location mention. Even if two bacteria mentions are coreferential, only one of them can be involved in an event because the BB-event datasets annotate only one event for coreferential entities. This suggests that a single location mention is unlikely to be involved both in the intra-sentence and cross-sentence events at the same time.

Based on these observations, we select candidate mentions for cross-sentence events as follows. For a given bacteria mention, we select it as a candidate only if (1) it is found in a sentence that contains no location mention at all, or (2) it already has at least one intra-sentence relation with any location. In other words, we select it as long as it has a relation with any location mention that exists in the same sentence. For a given location mention, we select it unless it already has an event relation with any bacteria mention in the same sentence. This means that we extract cross-sentence events based on the results of intra-sentence extraction.

Once candidate mentions are selected based on these criteria, we start with bacteria mentions one by one, from top to bottom, searching for location mentions around them to form cross-sentence events. More specifically, for each candidate bacteria mention, we examine candidate location mentions one by one within the sliding context window of a fixed size. For each candidate location mention, we check whether it can have a cross-sentence relation with the given bacteria mention based on trigger-based inference in a way similar to long-range intra-sentence extraction. In this process, each context trigger is linked to only one bacteria mention closest to it (in terms of the number of words and sentences between them). Note that aside from using the context window of a fixed size, we also make sure that it does not include other previous or following bacteria mentions appearing in a document, but includes the only bacteria mention within it, in order to maintain precision in cross-sentence inference. Thus, our system does not attempt to find cross-sentence events for bacteria mentions that occur with other bacteria mentions in the same sentence.

**Fig. 6:**
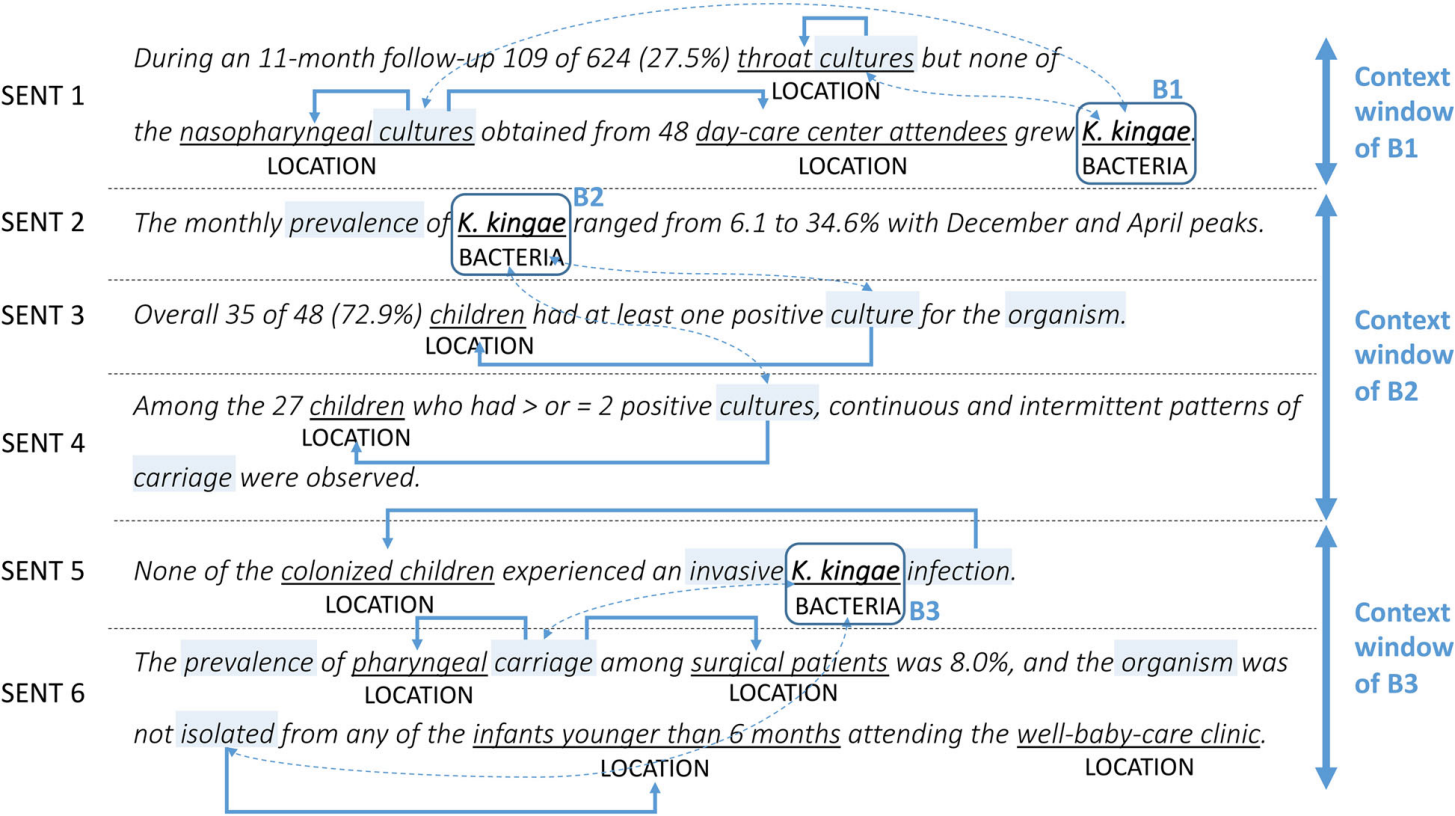
Example of the mapping between context triggers and bacteria mentions, and of linking each trigger to location mentions. Blue-shaded words such as *cultures* and *prevalence* are context triggers. Dashed curved arrows are the mappings between context triggers and bacteria mentions within the context window of each bacteria mention. Solid curved arrows connecting context triggers to location mentions are intra-sentence relations between them. Solid vertical arrows on the right indicate the sliding context window (i.e., sentence range) of each of the three bacteria mentions (B1, B2, and B3)

Figure 6 illustrates how context windows are established for each bacteria mention and how cross-sentence events are extracted based on the intra-sentence relations between context triggers and location mentions. For example, the cross-sentence relation between *K. kinage* (B3) in sentence 5 and *pharyngeal* in sentence 6 is extracted based on the mapping between *carriage* (the trigger) and B3, and the intra-sentence relation between *carriage* and *pharyngeal*. Note that the context window of each bacteria mention is created in such a way that it does not contain other bacteria mentions. For example, the context window of *K. kinage* (B1) contains only one sentence (i.e., sentence 1) because there is another bacteria mention *K. kinage* (B2) in the subsequent sentence (i.e., sentence 2).

### Dealing with hypothesis, negation, and research goals

The BB-event annotation guidelines state that the corpus does not annotate any working hypothesis as an event. However, the corpus does not explicitly mark such information, and the annotation guidelines do not specifically describe how to identify working hypotheses. This complicates the overall task further because it is challenging to determine which part of statements is hypothetical or not, even by humans. Our analysis reveals that diverse types of relevant linguistic modality are reflected in the event annotations, such as negation and speculation, which significantly affect the automatic identification of events. This type of linguistic information has not been investigated in detail in previous work on the BB-event task for two reasons. First, sophisticated detection of such information (e.g., identifying the scope of negation) requires another line of extensive research. Second, supervised learning models are generally assumed to capture such information using surface tokens and part-of-speech information. We do not fully address this issue in this paper as it is considered beyond the scope of this work. Nonetheless, we found that a simple analysis of the syntactic relationship between entity mentions and keywords that indicate linguistic modalities such as a working hypothesis significantly improves the performance of event extraction. In particular, we see that when entity mentions (including context triggers) are syntactically dominated by modality keywords, those entities are unlikely to form an event. The two consecutive sentences in Example 15 demonstrate an example of this case. They contain one bacteria mention, three location mentions, and four occurrences of context triggers underlined (*maturation*, *adhesion*, and *infection*). Here, bacteria mention *S. marcescens MG1* is annotated to have event relations with two occurrences of location mention *biofilm* (one for intra-sentence and the other for cross-sentence). However, it does not have an event relation with the third location mention *abiotic and biotic surfaces*, despite the same trigger (*adhesion*) repeatedly used for both the second and the third location mentions. Again, we see that this is because the second occurrence of *adhesion* lies in the scope of a working hypothesis, created by “*determine whether*”. Our analysis revealed that some verbs and their nominalized form, such as *determine* and *determination*, are used to create the scope of linguistic modalities that impact event extraction, as shown in this example. Example 15. Linguistic modality and event relation [PMID: 17237163]

- *In previous studies of* [*S. marcescens MG1*] **BACTERIA***, we showed that*[*biofilm*] **LOCATION***maturation* …
- *Because of the importance of *adhesion* in initiating*[*biofilm*] **LOCATION***formation and infection, the primary goal of this study was ****to determine whether**** QS is important in adhesion to both*[*abiotic and biotic surfaces*] **LOCATION** …

We employ the following three methods for detecting such linguistic modalities to filter out given entities (or triggers), thus preventing them from being involved in events. Although this is a simple and limited approach, it is found to boost the overall performance by raising precision without loss of recall, as detailed in the “Results” section.

1. In order to detect the hypothetical statements, we use the same list of keywords as used to filter out non-trigger expressions in the trigger collection process, such as *study*, *analysis*, *examination*, and *determination*, together with their verb forms, such as *analyze*, *examine*, and *determine*. When these modality keywords are found in a given sentence, we examine its dependency structure produced by the dependency parser and filter out entities and triggers that are syntactic descendants of the keywords (e.g., the trigger *adhesion* is a syntactic descendant of *determine* in the second sentence of Example 15).
2. We filter out entities and triggers if they are within *if*-clauses or *whether*-clauses.
3. In order to deal with negations, we filter out all the entities and triggers that are directly modified by any of the three negation expressions *not*, *no* and *none of*, such as “*None of BACTERIA is isolated from LOCATION*”. We also filter out entities and triggers if they are modified by any predicates (verbs and adjectives) that are negated via *not*, such as “*BACTERIA is not isolated from LOCATION*”.

We evaluated the proposed methods with the official BB-event evaluation benchmark. Experimental data, settings, and results are detailed in the “Results” section. Error analyses and remaining issues are presented in the “Discussion” section.

## Data Availability

The datasets used in this study are publicly available at the official website of BioNLP Shared Task 2016 http://2016.bionlp-st.org. The source codes used in the experiments are available at https://github.com/jwchung-lab/bionlp-bb-ctrigger.

## Abbreviations

BB-event: Bacteria-Biotope event
CDR: Chemical-disease relation
LSTM: Long short-term memory
SVM: Support vector machine
